# Genetic Deficiency of Hyaluronan Synthase 2 in the Developing Limb Mesenchyme Impairs Postnatal Synovial Joint Formation

**DOI:** 10.3390/biomedicines13061324

**Published:** 2025-05-28

**Authors:** Yingcui Li, Alexander Tress, Peter Maye, Kemar Edwards, Asiona Findletar, Nathaniel A. Dyment, Yu Yamaguchi, David W. Rowe, Gengyun Le-Chan, Sunny S. K. Chan, Kevin W.-H. Lo

**Affiliations:** 1 Department of Biology, College of Arts and Sciences, University of Hartford, 200 Bloomfield Avenue, West Hartford, CT 06117, USA; tress@hartford.edu (A.T.); kemaredwards82@gmail.com (K.E.); 123celestemiseh@gmail.com (A.F.); gle@hartford.edu (G.L.-C.); 2 Center for Regenerative Medicine and Skeletal Development, University of Connecticut Health Center, Farmington, CT 06032, USA; pmaye@uchc.edu (P.M.); drowe@uchc.edu (D.W.R.); 3 Department of Orthopedic Surgery, University of Pennsylvania, Philadelphia, PA 19104, USA; dyment@pennmedicine.upenn.edu; 4 Human Genetics Program, Sanford Burnham Prebys Medical Discovery Institute, La Jolla, CA 92037, USA; yyamaguchi@sbpdiscovery.org; 5 Department of Pediatrics, University of Minnesota, Minneapolis, MN 55455, USA; sschan@umn.edu; 6 Department of Medicine, University of Connecticut Health Center, 263 Farmington Ave, Farmington, CT 06030, USA

**Keywords:** synovial joint formation, limb development, cartilage homeostasis, hyaluronan, hyaluronan synthase 2 (*Has2*)

## Abstract

Hyaluronan, a key component of the extracellular matrix, plays a crucial role in joint development and maintenance. In order to determine the role of hyaluronan function in joint development and homeostasis, conditional loss-of-function experiments of Hyaluronan Synthase 2 (*Has2*) were carried out in mice. *Has2* depletion in limb mesenchymal cells led to severely shortened limbs with appendicular joints that are deformed, decreased proteoglycan content as characterized by Safranin-O staining, and severely pitted epiphyseal ends of long bones and deformed joints as viewed by micro-CT reconstructions. The embryonic deletion of *Has2* in mesoderm mesenchyme of limbs by Prx1-Cre confirmed its involvement in joint development, while in situ hybridization and hyaluronan staining confirmed *Has2* expression and abundant accumulation of hyaluronan in the onset of joint formation, the joint interzone. These findings position *Has2* as the main hyaluronan-making enzyme in articular cartilage and highlight its essential function in joint formation and retention of proteoglycans of the extracellular matrix of the cartilage.

## 1. Introduction

Synovial joints play a crucial role in movement and weight bearing. The epiphyseal ends of endochondral bones are covered by articular cartilage, a hyaline-based cartilage present within synovial joints [1,2]. The development of synovial joints is initiated by a specialized group of skeletal progenitors located in a distinct region of the developing endochondral bones known as the joint interzone [1,3]. Formation of the interzone occurs when differentiated chondrocytes lose their chondrocyte-like characteristics, alter their fate as growth plate chondrocytes, and acquire a new fate as progenitors of the synovial joint [1,2,3,4,5]. The embryonic development of synovial joints starts with interzone formation and cavitation, where condensed mesenchymal cells create a joint cavity. During postnatal maturation, cartilage undergoes structural transformation into three distinct zones known as superficial, intermediate, and deep zones, which exhibit unique cell and matrix characteristics necessary for smooth joint movement and mechanical stress resistance [6].

In articular cartilage, hyaluronan, a large glycosaminoglycan (GAG), represents an abundant component of the extracellular matrix [7,8,9]. Research has demonstrated the critical role of hyaluronan in synovial joint lubrication and overall joint health [9,10,11,12]. Conversely, clinical studies have shown that levels of hyaluronan are decreased in osteoarthritic cartilage [13,14,15]. Thus, an inverse correlation exists between hyaluronan abundance and joint health.

To better understand the function of hyaluronan, genetic mouse models for the *Hyaluronan Synthase* (*Has*) genes have been developed [16,17,18]. These genes *(Has1*, *Has2*, *Has3)* encode enzymes responsible for the production of hyaluronan. *Has2* is the primary isoform expressed during endochondral bone formation and is responsible for hyaluronan production during skeletal development [8,19,20]. Prior studies on conditional or constitutive *Has2* loss of function studies have highlighted its critical role in skeletal development [16,21]. Specifically, when *Has2* was conditionally inactivated in using *Prx1-Cre* mice, defects were observed in joint formation, growth plate organization and gene expression of *Ihh* and *Col10,* and formation of the secondary ossification center [16]. While previous studies have demonstrated the critical role of *Has2* in embryonic skeletal development, the long-term impact of its disruption on postnatal joint development and homeostasis remains unclear. This study explores the hypothesis that Hyaluronan Synthase 2 (*Has2*) is crucial for synovial joint development throughout the embryonic stage and into postnatal life. Specifically, we investigate that the conditional inactivation of *Has2* in limb mesenchyme leads to abnormal joint development and long-term cartilage abnormalities.

In this report, we focus on the role of *Has2* and hyaluronan in postnatal joint formation using the *Prx1-Cre* mice model.

*Prx1* is a transcription factor essential for limb development, regulated by BMP and FGF pathways. Its limb-specific enhancer drives Cre recombinase in transgenic mice, enabling precise gene editing from embryonic day 9.5, with recombination complete by day 10.5. This tool allows spatially and temporally controlled studies of limb development. The *Prx1-Cre* model is used to conditionally inactivate *Has2* in limb mesenchyme, aiding research on its developmental role in both forelimbs and hindlimbs [16,22,23,24].

Our results reveal that the conditional loss of *Has2* at embryonic ages has long-term postnatal consequences, including diminished proteoglycan levels and progressive joint (postnatal maturation). The synthesis of hyaluronan via *Has2* is essential for preserving extracellular matrix stability and protecting against joint degenerative conditions [25,26]. This work provides novel evidence for the essential role of hyaluronan through the long-term consequences of embryonic hyaluronan loss on joint health and postnatal joint integrity.

## 2. Material and Methods

### 2.1. Mice

All animal experiments were performed under an approved protocol by the Institutional Animal Care and Use Committee at the University of Hartford (IACUC# UH-2023-01). We generated phenotypically normal wildtype *Has2^fl/fl^* mice with a functional conditional allele by targeting exon 2 (containing the start codon and transmembrane domains) using loxP-flanked Neo/DTA cassettes in 129SvJ-derived clones as described [16]. *Prx1-Cre* mice [22] were generously provided by Dr. Clifford Tabin. It was generated using a limb-specific enhancer to drive Cre recombinase in limb bud mesenchyme by 9.5 dpc, enabling targeted gene deletion for studying limb-patterning genes during limb development. All mice were housed in a room maintained on a 12:12 h light/dark cycle with controlled temperature and humidity and fed a standard rodent diet.

### 2.2. Mouse Crosses

*Has2^fl/fl^* homozygous females were intercrossed with *Prx1-Cre* male mice. All offspring showed normal phenotypes. *Prx1-Cre*; *Has2^fl/+^* heterozygous males from the offspring were intercrossed with *Has2^fl/fl^* to generate mutant *Prx1-Cre*; *Has2^Δ/Δ^* mice. Age-matched littermates of *Has2^fl/fl^* served as wild-type embryo/mouse controls for mutant *Prx1-Cre; Has2*^Δ/Δ^ mice. Logan et al. reported that Prx1-Cre can exhibit germline recombination in offspring of female *Prx1-Cre* carriers but not in offspring of male carriers, indicating sex-dependent leakiness [22]. Only male mice were used in the subsequent study due to potential Cre leaky in females, which could affect the interpretation of genotype effects.

### 2.3. Tissue Processing for Histology and Staining

Tissues were fixed with 4% paraformaldehyde for 2–4 h for embryonic day 14.5 (E14.5) and 2–3 days for post-natal tissues collected at neonatal day 3 (P3) and day 21 (P21). Postnatal tissues were decalcified with 14% EDTA for 3–5 days before paraffin embedding. Moreover, 8 μm paraffin sections were collected.

### 2.4. In Situ Hybridization

In situ hybridization was carried out on 8 μm paraffin tissue sections using a ^33^P labeled probe or digoxigenin-labeled probe for *Has2* as previously described [16]. The *Has2* probe was a 752 bp cDNA probe extended from 1461–2212 at the 3′ end of the cDNA derived from XmaI/EcoRI digestion of a pCI-Neo vector containing full-length mouse *Has2*. For spatial mapping, dark-field silver grains overlaid onto corresponding Hematoxylin-stained bright-field images in Photoshop version 26.6.1.

### 2.5. Histochemical Staining

Histochemical staining was used to detect the amount of hyaluronan accumulation in embryonic joints and adult joints by 2 μg/mL biotinylated hyaluronan-binding protein (HABP) overnight at 4 °C (Sigma-Aldrich, Saint Louis, MO, USA) as previously described [8,16]. Negative HABP staining control sections were pretreated for 2 h at 55 °C with 200 TRU/mL of *Streptomyces hyalurolyticus* hyaluronidase (Sigma-Aldrich, Saint Louis, MO, USA), which specifically degrades HA.

Histochemical staining with Safranin-O (Sigma-Aldrich, Saint Louis, MO, USA) with fast green (Sigma-Aldrich, Saint Louis, MO, USA) counterstaining was used to evaluate cartilage proteoglycan content and chondrocyte cellular morphology [16]. Paraffin sections were stained with Weigert’s Iron Hematoxylin and 0.02% aqueous Fast Green, followed by rinsing with 1% acetic acid and staining with 0.1% aqueous Safranin O. Alcian Blue and Alizarin Red staining were used for whole mount skeletal staining to visualize cartilage and mineralized bone, respectively [16]. Briefly, embryos and neonatal mice collected were fixed in 95% EtOH for 1 week, stained in 0.015% Alcian Blue (Sigma-Aldrich, Saint Louis, MO, USA) in 95% EtOH Glacial Acetic Acid for 3–4 days, and 75 µg/mL Alizarin Red (Sigma-Aldrich, Saint Louis, MO, USA) in 1% KOH for 1 day. Tissues were cleared in graded KOH and photography in 75% glycerol [16,27].

### 2.6. Microcomputed Tomography

The architecture of joints from both normal wildtype *Has2^fl/fl^* littermate and mutant *Prx1-Cre; Has2*^Δ/Δ^ were compared using 3-dimensional (3D) microcomputed tomography (µCT) imaging (μCT40, ScanCo Medical AG, Bassersdorf, Switzerland) at UConn Health [28]. The limbs were scanned in water using high-resolution settings, with an energy level of 55 kVp and an intensity of 145 μA, at an integration time of 500 ms. The images were reconstructed and calibrated to an isotropic voxel size of 8 mm^3^.

## 3. Results

### 3.1. Genetic Loss of Has2 in the Limb Bud Mesenchyme Causes Joint Defects

**Figure 1:**
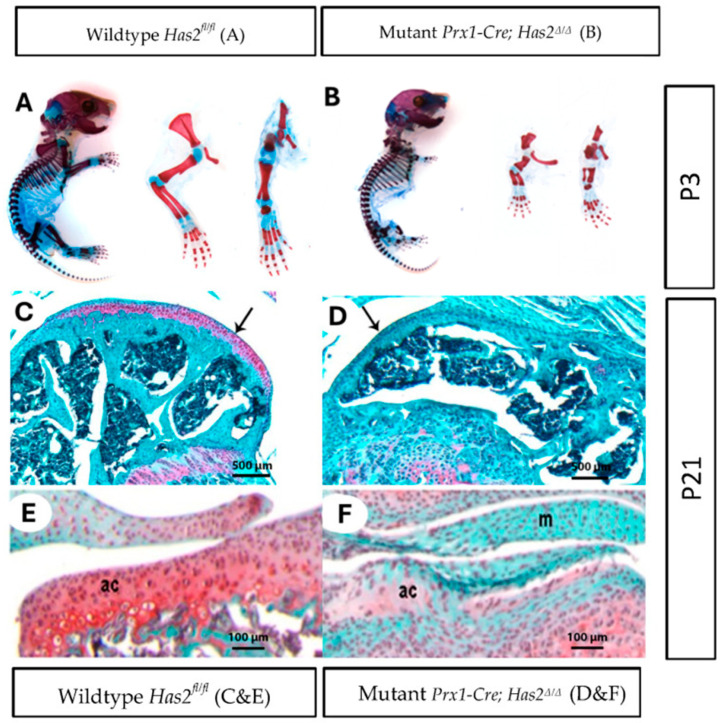
Limb bud mesenchyme-specific knockout of *Has2* induced severe joint defects and impaired the differentiation and formation of articular cartilage. (**A**,**B**) Whole mount staining of cartilage and mineralized bone with Alcian Blue and Alizarin Red, respectively, in neonatal day 3 (P3) (**A**) wildtype *Has2^fl/fl^* and (**B**) mutant *Prx1-Cre*; *Has2^Δ/Δ^* mice. Left to right: whole body, forelimb, hindlimb (0.63×). (**C**–**F**) Paraffin sections of the joint surface of 3-week-old (**C**) wildtype *Has2^fl/fl^* and (**D**) mutant *Prx1-Cre*; *Has2^Δ/Δ^* femurs stained with Safranin-O (red) for proteoglycan accumulation (counterstained with fast green). The wildtype *Has2^fl/fl^* femur is capped by a thick layer of articular cartilage, which stains intensely with Safranin-O, while the hyaluronan-deficient femur is capped by a thin layer of tissue that does not stain with Safranin-O, indicating normal articular cartilage is not present (4×) (pointed by black arrow). (**E**,**F**) Sections of the tibia joint surface of 3-week-old (P21) (**E**) wildtype *Has2^fl/fl^* and (**F**) mutant *Prx1-Cre*; *Has2^Δ/Δ^* mice stained with Safranin-O/fast green. In the wildtype *Has2^fl/fl^* tibia, a layer of articular cartilage (ac) with round chondrocytes and abundant Safranin-O matrix is present, but the joint surface in the hyaluronan-deficient tibia is covered by a highly cellular tissue with flattened cells and little or no Safranin-O staining, indicating initial articular cartilage differentiation is impaired. Menisci (M) in the hyaluronan-deficient joint also showed no or very little Safranin-O staining. This underscores the importance of *Has2* in joint development for different types of chondrocytes (20×).

To investigate the effect of *Has2* on synovial joint formation, we examined *Prx1-Cre; Has2^Δ/Δ^* mice, in which *Has2* was conditionally inactivated by *Prx1-Cre*. In these mice, *Has2* was removed in early limb bud mesenchyme prior to the onset of mesenchymal condensations and the formation of joint interzones. Similar to previous reports [16], the appendicular skeleton of mutant *Prx1-Cre; Has2^Δ/Δ^* mice was severely compromised at neonatal day 3 (P3). The forelimbs and hindlimbs of mutant *Prx1-Cre; Has2^Δ/Δ^* mice were dramatically shorter than their wildtype *Has2^fl/fl^* littermates. The distal limb region and paws were also shortened (Figure 1A,B). In all sixteen P3 mutant *Prx1-Cre; Has2^Δ/Δ^* mice examined, the limbs displayed consistent phenotypic changes, with shortening of the skeletal elements. The skeletal elements of P3 mutant *Prx1-Cre; Has2^Δ/Δ^* from proximal to distal—stylopod, zeugopod, and autopod—were shortened by 58%, 60%, and 35%, respectively, in the forelimbs and by 52%, 58%, and 32%, respectively, in the hindlimbs comparing to the wildtype *Has2^fl/fl^* mice.

Next, we determined the long-term consequences of *Prx1-Cre*-mediated inactivation of *Has2* in 3-week-old animals. Safranin-O staining on joint tissue sections revealed remarkable differences in proteoglycan content within the cartilage matrix. Whereas the wildtype *Has2^fl/fl^* femurs were capped by a thick layer of Safranin-O-positive articular cartilage, *Has2* deficient femurs were capped by a thin layer of Safranin-O-negative tissue (Figure 1C,D), black arrows demarcate articular surface). Not surprisingly, significant reductions in cartilage proteoglycan content were also observed in mutant *Prx1-Cre; Has2^Δ/Δ^* tibia (Figure 1E,F). Moreover, *Has2* conditional mutants appeared to have less cartilage matrix production and denser intercellular distances among chondrocytes within the matrix.

### 3.2. Has2 Is Essential for Proper Bone Formation at Synovial Joints

**Figure 2:**
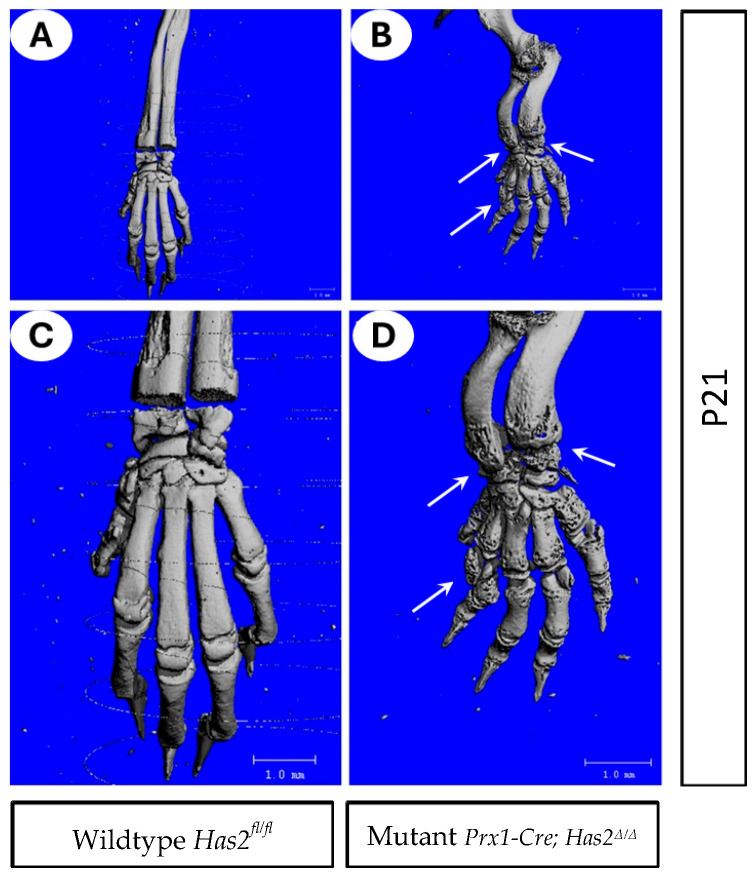
Mutant *Prx1-Cre; Has2^Δ/Δ^* mice exhibited improper bone formation (**A**–**D**) 3-D µCT images of forelimb and wrist joints of 3-week-old (P21) (**A**,**C**) wildtype *Has2^fl/fl^* and (**B**,**D**) mutant *Prx1-Cre; Has2^Δ/Δ^* mice. (**C**,**D**) are magnified images of (**A**,**B**), respectively. Joint spaces were intact in wild-type littermate (**A**,**C**) but showed erosion of bone and heavily eroded wrist (arrows in (**B**,**D**)). Arch-like deformation of radius and ulna was also observed in the mutant.

We further examined the forelimb and wrist joints of wildtype *Has2^fl/fl^* and mutant *Prx1-Cre; Has2^Δ/Δ^* mice at 3 weeks of age using 3-D µCT (Figure 2A–D). Remarkably, *Has2* conditional mutants showed severe erosion at the epiphyseal ends of bones as evidenced by extensive pitting (Figure 2B,D). Arch-like deformations of the radius and ulna were also observed in *Has2* mutants (Figure 2B,D).

### 3.3. Has2 and Hyaluronan Are Localized at the Joint Interzone

**Figure 3:**
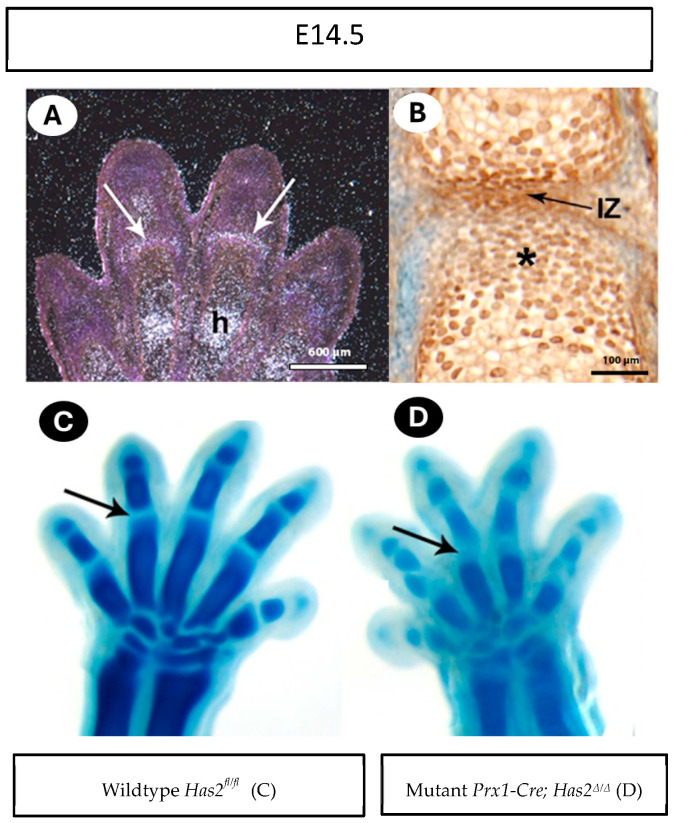
*Has2* and hyaluronan are localized at the joint interzone. (**A**) At embryonic day 14.5 (E14.5), *Has2* mRNA is present in joint interzones (arrows) and chondrocytes undergoing hypotrophy (h). (**B**) HABP staining shows abundant hyaluronan in the interzone (IZ) and abundant in the cartilage cells adjacent to IZ (asterisk). (**C**,**D**) E14.5 wildtype *Has2^fl/fl^* and mutant *Prx1-Cre; Has2^Δ/Δ^* paws stained with Alcian blue. The wildtype *Has2^fl/fl^* joint interzones are narrow (arrow in (**A**)), while *Has2*-deficient interzones are wide (arrow in (**B**)).

To provide additional evidence for the early role of *Has2* and hyaluronan in joint formation, *Has2* expression and hyaluronan production were examined in E14 embryos in phalangeal joints. In situ hybridization of *Has2* mRNA revealed selective expression at the joint interzone (Figure 3A, white arrows). We further observed the abundant accumulation of hyaluronan in the interzone, as well as in the surrounding chondrocytes (Figure 3B; asterisk) by immunostaining of hyaluronan with biotinylated hyaluronan-binding protein (HABP) (Figure 3B, black arrow).

The presence of *Has2* and hyaluronan in the joint interzone suggests a direct and early role for hyaluronan in joint formation (Figure 3A). Indeed, Alcian blue staining of the paws of E14.5 embryos revealed that the interzone regions of mutant *Prx1-Cre; Has2^Δ/Δ^* mutants were wider and less defined than their wildtype *Has2^fl/fl^* littermates (Figure 3C,D, arrows).

## 4. Discussion

Synovial joints are essential for our mobility and diseases that impact joint function are closely tied to quality of life and our overall health. Diseases of the joint include congenital conditions that can result in joint deformity resulting in premature malfunction, sometimes affecting one specific joint type over another. The genetic causes of synovial joint birth defects and the signaling molecules required for joint homeostasis after birth remain poorly understood.

Hyaluronan is a large glycosaminoglycan molecule abundant in the cartilage matrix. Traditionally, hyaluronan function in cartilage matrix has been believed as a space-filling molecule, where it serves a structural role through binding to other cartilage matrix molecules by forming large proteoglycan aggregates during limb development. However, emerging evidence suggests that hyaluronan also plays a signaling role during joint formation. For instance, overexpression of hyaluronan in embryonic chicken limb mesenchymal cells results in missing or malformed joints [8], whereas loss of hyaluronan leads to severely shortened limbs with defective synovial joint cavities [16]. These findings indicate that hyaluronan is not only a passive ECM component but also a key regulator of joint morphogenesis. Our previous study found that *Has2* is expressed in the distal posterior subridge mesoderm and AER during early limb formation, as well as in digit tips but not precartilage or interdigital mesoderm. While HA downregulation enables precartilage condensation and cartilage differentiation, later *Has2* and HA accumulate in joint-forming interzones and in hypertrophic chondrocytes, emphasizing the importance of HA in interzone formation and cartilage-to-bone transition. Studies using fate-mapping have shown all of the structures of the adult synovial joint, including the articular cartilage, as well as non-chondrogenic tissue such as ligament and synovium, arise from these joint progenitor cells [1,29]. Thus, the joint interzones contain a mixed population of progenitors with distinct chondrogenic or non-chondrogenic fates. In mutants lacking hyaluronan, interzone regions were wider and less defined because hyaluronan loss disrupts the balance between chondrogenic and non-chondrogenic fates. Without hyaluronan, joint progenitor cells may favor non-chondrogenic differentiation (e.g., ligaments) over articular cartilage formation. This shift leads to less distinct interzones with mixed cell fates and impaired tissue boundaries. The absence of Alcian blue staining in the expanded interzone, suggesting no cartilage formation, further confirms the altered fate specification. The absence of hyaluronan leads to an increase in interzone space because hyaluronan normally provides hydration and swelling pressure that helps maintain tissue cohesion and structure; without it, the matrix becomes disorganized and less compact [30,31]. Hyaluronan is a major component of the cartilage matrix, along with aggrecan and other proteoglycans. It forms a hydrated network in cartilage tissues. Other molecules, such as collagens that can contribute to interzone structure and are often crosslinked with proteoglycans for proper extracellular matrix (ECM) organization, do not possess the hydrophilic and space-filling qualities of hyaluronan. Additionally, hyaluronan plays a key role in establishing proteoglycan aggregates, which are essential components of the cartilage extracellular matrix. The primary proteoglycan aggregate in cartilage consists of aggrecan, hyaluronan, and link protein. Due to these functions, hyaluronan’s unique contribution cannot be replaced in preserving proper tissue structure and spacing for normal joint formation [32].

While hyaluronan contributes to the extracellular matrix of articular cartilage, we demonstrate that *Has2* plays a critical role in both embryonic and postnatal joint development. Our data show that loss of *Has2* results in important long-term impairments of joint health. These observations indicate that *Has2* and hyaluronan are dynamic mediators of cartilage repair and degeneration. This work reveals novel information on the central function of *Has2* and hyaluronan in embryonic and postnatal joint development. These data provide a novel insight into how genetic loss of *Has2* has far-reaching, long-term impacts on cartilage health and joint function. The correlation between articular cartilage loss of *Has2* and diminished levels of proteoglycan and the erosion of bone on the epiphyseal edges underscores the role of *Has2*-mediated hyaluronan synthesis in maintaining the ECM of articular cartilage. It provides a new phenotype that underscores the critical role of *Has2* in the dynamic maintenance and repair of synovial joints.

Using the *Prx1-Cre* genetic mouse model, we show that deletion of *Has2* from embryonic limb mesenchymal cells results in rapid cartilage degradation, suggesting that hyaluronan synthesis is essential for continuous cartilage maintenance. Consistent with what has been previously reported [15], the loss of *Has2* in limb bud mesenchyme with *Prx1-Cre* resulted in defective joint formation. Interestingly, while changes in joint formation were histologically apparent at the earliest stages of joint formation, the long-term consequences on articular cartilage formation and joint health have not been reported. Examination of 3-week-old femurs and tibias from mutant *Prx1-Cre; Has2^Δ/Δ^* mice, when all types of chondrocytes are already differentiated, indicated a considerable drop in cartilage proteoglycan content as determined by Safranin-O staining. Moreover, examination of forelimbs by 3D-µCT at 3 weeks of age not only revealed bone deformities but extensive pitting, suggesting extensive erosion has occurred in focal areas at the epiphyseal ends of long bones.

Hyaluronidases and matrix metalloproteases (MMP) are known to degrade hyaluronan [33,34,35], but the continued synthesis of hyaluronan by *Has2* has limited our ability to determine which protease(s) are primarily responsible. Certainly, developing a deeper understanding of the dynamic turnover of cartilage extracellular matrix molecules has important implications for diseases like osteoarthritis. Observing the marked changes that have occurred in the cartilage extracellular matrix due to the loss of *Has2* and hyaluronan will clearly need to be further investigated to gain a better understanding of its mechanistic role in cartilage. Further studies investigating the involvement of MMPs and inflammatory cytokines in *Has2*-deficient models may provide mechanistic insights into how hyaluronan depletion contributes to cartilage breakdown and joint pathology.

The results of our study provide essential insights into the development mechanisms of osteoarthritis (OA). OA develops through articular cartilage degradation, which occurs alongside diminished hyaluronan concentrations in the extracellular matrix. The joint pathology displayed in *Has2*-deficient mice, which includes eroded cartilage and diminished proteoglycan levels, mirrors the typical OA disease characteristics. The results demonstrate that *Has2* activity and hyaluronan production help maintain cartilage health by preventing its degradation. Our findings demonstrate that the impairment in *Has2* function could increase OA risk while identifying *Has2* as a potential early-stage joint disease biomarker. The development of treatments that target *Has2* signaling pathways offers a potential new therapeutic strategy for OA by both restoring hyaluronan synthesis and maintaining cartilage integrity. Researchers need to investigate drug treatments that can boost *Has2* activity or hyaluronan production to stop OA from advancing.

In conclusion, the central role of *Has2* and hyaluronan in joint health makes them prime candidates for novel clinical interventions. Such research not only broadens our knowledge of joint biology but also opens a better way to treat and prevent chondrocyte diseases.

## Data Availability

The original contributions presented in this study are included in the article. Further inquiries can be directed to the corresponding authors.
