# Correction: Li et al. Genetic Deficiency of Hyaluronan Synthase 2 in the Developing Limb Mesenchyme Impairs Postnatal Synovial Joint Formation. *Biomedicines* 2025, *13*, 1324

**DOI:** 10.3390/biomedicines14010004

**Published:** 2025-12-19

**Authors:** Yingcui Li, Alexander Tress, Peter Maye, Kemar Edwards, Asiona Findletar, Nathaniel A. Dyment, Yu Yamaguchi, David W. Rowe, Gengyun Le-Chan, Sunny S. K. Chan, Kevin W.-H. Lo

**Affiliations:** 1 Department of Biology, College of Arts and Sciences, University of Hartford, 200 Bloomfield Avenue, West Hartford, CT 06117, USA; tress@hartford.edu (A.T.); 123celestemiseh@gmail.com (A.F.); gle@hartford.edu (G.L.-C.); 2 Center for Regenerative Medicine and Skeletal Development, University of Connecticut Health Center, Farmington, CT 06032, USA; pmaye@uchc.edu (P.M.); drowe@uchc.edu (D.W.R.); 3 Department of Orthopedic Surgery, University of Pennsylvania, Philadelphia, PA 19104, USA; dyment@pennmedicine.upenn.edu; 4 Human Genetics Program, Sanford Burnham Prebys Medical Discovery Institute, La Jolla, CA 92037, USA; yyamaguchi@sbpdiscovery.org; 5 Department of Pediatrics, University of Minnesota, Minneapolis, MN 55455, USA; 6 Department of Medicine, University of Connecticut Health Center, 263 Farmington Ave, Farmington, CT 06030, USA

## Error in Figure

In the original publication [1], there was a mistake in Figure 1B as published. We recently discovered that one of the images in Figure 1B of our published article was accidentally taken from one of our previous publications during figure assembly. The corrected version of Figure 1B appears below. The authors state that the scientific conclusions are unaffected. This correction was approved by the Academic Editor. The original publication has also been updated.

## Figures and Tables

**Figure 1:**
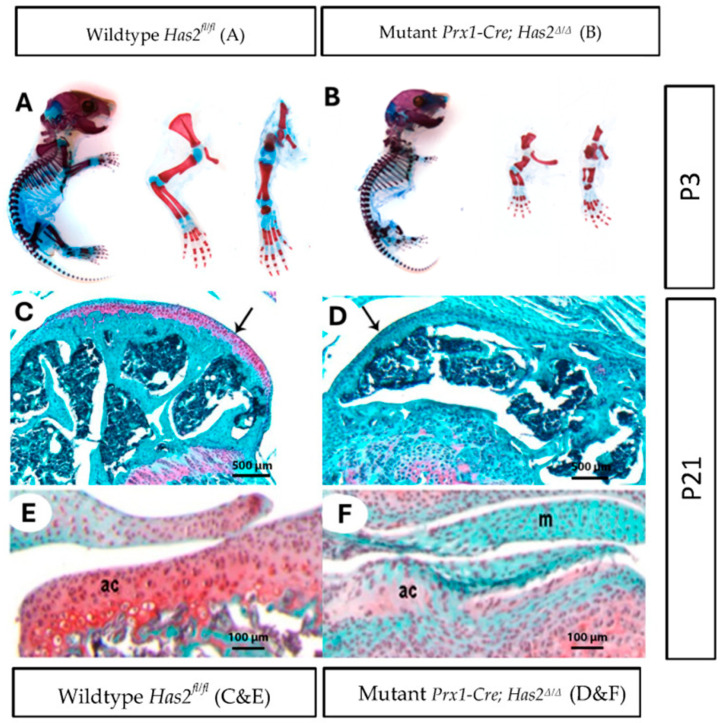
Limb bud mesenchyme-specific knockout of *Has2* induced severe joint defects and impaired the differentiation and formation of articular cartilage. (**A**,**B**) Whole mount staining of cartilage and mineralized bone with Alcian Blue and Alizarin Red, respectively, in neonatal day 3 (P3) (**A**) wildtype *Has2^fl/fl^* and (**B**) mutant *Prx1-Cre*; *Has2^Δ/Δ^* mice. Left to right: whole body, forelimb, hindlimb (0.63×). (**C**–**F**) Paraffin sections of the joint surface of 3-week-old (**C**) wildtype *Has2^fl/fl^* and (**D**) mutant *Prx1-Cre*; *Has2^Δ/Δ^* femurs stained with Safranin-O (red) for proteoglycan accumulation (counterstained with fast green). The wildtype *Has2^fl/fl^* femur is capped by a thick layer of articular cartilage, which stains intensely with Safranin-O, while the hyaluronan-deficient femur is capped by a thin layer of tissue that does not stain with Safranin-O, indicating normal articular cartilage is not present (4×) (pointed by black arrow). (**E**,**F**) Sections of the tibia joint surface of 3-week-old (P21) (**E**) wildtype *Has2^fl/fl^* and (**F**) mutant *Prx1-Cre*; *Has2^Δ/Δ^* mice stained with Safranin-O/fast green. In the wildtype *Has2^fl/fl^* tibia, a layer of articular cartilage (ac) with round chondrocytes and abundant Safranin-O matrix is present, but the joint surface in the hyaluronan-deficient tibia is covered by a highly cellular tissue with flattened cells and little or no Safranin-O staining, indicating initial articular cartilage differentiation is impaired. Menisci (M) in the hyaluronan-deficient joint also showed no or very little Safranin-O staining. This underscores the importance of *Has2* in joint development for different types of chondrocytes (20×).
